# Characterization of a Vibriophage Infecting Pathogenic *Vibrio harveyi*

**DOI:** 10.3390/ijms242216202

**Published:** 2023-11-11

**Authors:** Yingying Li, Huayi Yun, Ruo Chen, Nianzhi Jiao, Qiang Zheng, Yunlan Yang, Rui Zhang

**Affiliations:** 1 State Key Laboratory of Marine Environmental Science, College of Ocean and Earth Sciences, Xiamen University, Xiamen 361102, China; liyinging94@outlook.com (Y.L.); yunhy_1@126.com (H.Y.); mjq964@alumni.ku.dk (R.C.); jiao@xmu.edu.cn (N.J.); zhengqiang@xmu.edu.cn (Q.Z.); 2 Institute for Advanced Study, Shenzhen University, Shenzhen 518061, China; 3 Marine Biological Section, Department of Biology, University of Copenhagen, 3000 Helsingør, Denmark

**Keywords:** *Vibrio* phage, siphovirus, biological characteristics, genomic analysis, phage therapy

## Abstract

Bacterial diseases caused by *Vibrio* spp. are prevalent in aquaculture and can lead to high mortality rates among aquatic species and significant economic losses. With the increasing emergence of multidrug-resistant *Vibrio* strains, phage therapy is being explored as a potential alternative to antibiotics for biocontrol of infectious diseases. Here, a new lytic phage named vB_VhaS_R21Y (R21Y) was isolated against *Vibrio harveyi* BVH1 obtained from seawater from a scallop-farming area in Rongcheng, China. Its morphology, infection cycle, lytic profile, phage stability, and genetic features were characterized. Transmission electronic microscopy indicated that R21Y is siphovirus-like, comprising an icosahedral head (diameter 73.31 ± 2.09 nm) and long noncontractile tail (205.55 ± 0.75 nm). In a one-step growth experiment, R21Y had a 40-min latent period and a burst size of 35 phage particles per infected cell. R21Y was highly species-specific in the host range test and was relatively stable at pH 4–10 and 4–55 °C. Genomic analysis showed that R21Y is a double-stranded DNA virus with a genome size of 82,795 bp and GC content of 47.48%. Its high tolerance and lytic activity indicated that R21Y may be a candidate for phage therapy in controlling vibriosis in aquacultural systems.

## 1. Introduction

*Vibrio* spp. are Gram-negative, curved bacilli that occur naturally in marine, estuarine, and freshwater systems [1,2]. *Vibrio harveyi* (family Vibrionaceae, class Gammaproteobacteria) is a bacterial pathogen that seriously threatens marine fish and invertebrates, particularly penaeid shrimps, in the warmer waters of Asia, southern Europe, and South America [3,4]. This bacterium is notorious for causing a range of diseases, including luminous vibriosis in shrimp and eye lesions, blindness, gastroenteritis, muscle necrosis, skin ulcers, and tail rot disease in fish [4]. Diseases caused by *V. harveyi* have led to mass mortalities in both vertebrates and invertebrates, resulting in significant aquacultural losses. To minimize losses from bacterial infections, antibiotics are widely used in many countries. However, common use of these medications has led to the development of antibiotic-resistant bacteria, resulting in reduced effectiveness of these treatments over time [5,6]. Thus, shifting toward natural and probiotic treatments rather than antibiotic treatment is crucial [7,8]. As an effective alternative treatment, bacteriophages (phages) have been used as biocontrol agents to combat *V. harveyi* infections [8].

Phages are viruses that infect specific species or strains of bacteria. Contrary to the negative physiological effects of antibiotics on the host and the emergence of bacterial resistance, phages are eco-friendly and highly specific to their host bacterial strains [9,10]. Phages are categorized as lytic or temperate (lysogenic) based on their life strategies. Once a phage inserts its nucleic acid into the bacterial cytoplasm, the lytic phages highjack the host’s cellular machinery and consume its resources. The host is then killed when the phages release their progeny [11]. Alternatively, a temperate phage can become a prophage by inserting its genetic information into the genome of the host bacterium in the lysogenic cycle. The prophage’s genetic material is then passed to each daughter cell through cell division [12]. However, at some point, this passage of genetic material may be induced by chemicals, radiation, or carcinogens [13]. During excision of the phage DNA from the host chromosome, the host DNA may become incorporated into the phage DNA. Thus, lysogenic phages can facilitate the horizontal transfer of bacterial genes from one bacterium to another to enhance bacterial virulence, such as with the *Vibrio* temperate phage VHML [14,15]. Therefore, lytic phages are more suitable for phage therapy than are lysogenic phages. Furthermore, phages efficiently use lytic enzymes (endolysins) to directly target the bacterial cell wall [16,17] and are ubiquitous in all saltwater and freshwater environments, providing virtually unlimited sources of phages and lytic enzymes [18].

Both lytic and lysogenic bacteriophages have been isolated from China, India, Australia, Thailand, and many other countries to control *V. harveyi* infections in aquacultural products [19,20,21,22]. Several patents have been filed to use the phages VP4B, RDP-VP-19012, vB_KaS_PK22, vB_KaS_PK08, vB_VhaS-yong 1/2/3, VhaM_PcB-1G, and V-YDF132 to combat *V. harveyi* infections in China [23,24,25,26,27,28,29,30,31]. Despite the increasing number of isolated *Vibrio* phages, there is still much to learn about their genomes, ecology, evolution, and potential roles in bacteriophage therapy. In this study, we isolated and characterized the *V. harveyi* phage, vB_VhaS_R21Y (R21Y), and analyzed its morphology, infection kinetics, lytic profile, phage stability, and genetic content to identify its potential for future therapeutic applications.

## 2. Results

### 2.1. Biological Characterization of R21Y

**Figure 1:**
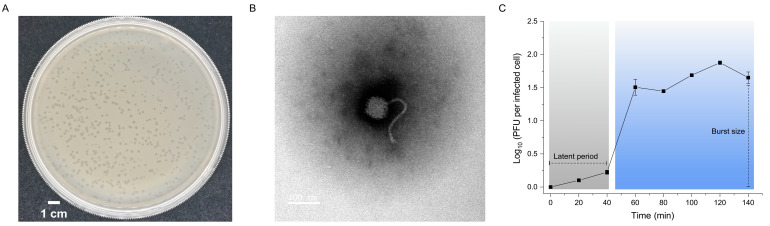
Isolation and biological features of *Vibrio* phage vB_VhaS_R21Y. (**A**) Plaques of vB_VhaS_R21Y formed on a lawn of *Vibrio harveyi* BVH1. (**B**) Transmission electron micrograph of vB_VhaS_R21Y. (**C**) One-step growth curve of vB_VhaS_R21Y. Error bars indicate standard deviations among triplicate samples.

Phage R21Y infecting *V. harveyi* BVH1 was isolated from seawater samples collected from the scallop-farming area of Ellen Bay in Rongcheng, Shandong, China (122°34′14.54″ W, 37°10′15.58″ E). R21Y produced clear and pinhole-type plaques with diameters of 0.69 ± 0.15 mm after incubation for 12 h at 28 °C (Figure 1A). Transmission electron microscopy showed that R21Y has an icosahedral capsid (diameter 73.31 ± 2.09 nm) and a long tail (205.55 ± 0.75 nm; Figure 1B), suggesting that R21Y belongs to the siphovirus morphotype.

The one-step growth curve (Figure 1C) shows that R21Y exhibited a latent period, defined as the duration between the irreversible binding of R21Y to the host cell and the onset of phage bursts, lasting for approximately 40 min. This was followed by a rise period lasting ~20 min, signifying the rapid release of phages from the infected host. The burst size of R21Y (i.e., the number of newly infectious phage particles produced per infected bacterial cell) was estimated to be approximately 35 phages.

Phage R21Y failed to infect any of the 40 tested *Vibrio* bacterial strains (including six pathogenic and 34 nonpathogenic strains), except for the original host *V. harveyi* BVH1 (Table 1). Thus, R21Y is likely a strain-specific phage with a high degree of specificity for its host strain.

### 2.2. Genomic Features of R21Y

R21Y has a circular double-stranded DNA genome of 82,795 bp and a G + C content of 47.48%. Analysis of the termini revealed that R21Y uses a headful (pac) packaging mechanism and contains redundant ends for circularizing the phage genome by recombination (Figure S1). Sequence analysis yielded 126 putative open reading frames (ORFs) with a total length of 82,325 bp (99.43% encoding percentage). BlastP analysis with amino acid sequences showed that 70 ORFs (55.56%) were the best matches to phage vB_VcaS_HC, which infects *V. campbellii*, with identities ranging from 84.2 to 100%. Forty-six ORFs (36.51%) had high identity with phage D4 (Virtus), matching 93.3–100%, and 8 ORFs (6.35%) showed similarities (40.3–98.5%) to other vibriophages, including 1, vB_ValS_PJ32, vB_VhaS-VHB1, 29Fa.3, and 1.215.A._10N.222.54.F7. The remaining ORFs (1.59%) were homologous to bacteria (Table S1).

**Figure 2:**
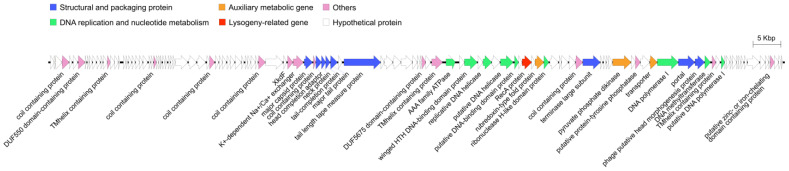
Full genome of phage vB_VhaS_R21Y. Putative open reading frames were assigned functional categories and are depicted as arrows.

The phage R21Y genes were categorized into six main modules: structural and packaging proteins, DNA replication and nucleotide metabolism, auxiliary metabolic genes, lysogeny-related genes, other functions, and hypothetical proteins (Figure 2). The phage structural and packaging proteins cluster contained nine ORFs, eight of which encoded typical structural proteins, including the major capsid protein (MCP; ORF 58), head completion adaptor (ORF 60), neck protein (ORF 61), tail completion protein (ORF 62), major tail protein (ORF 63), tail length tape measure protein (ORF 65), portal protein (ORF 105), putative head morphogenesis protein (ORF 106), and a gene encoding the terminase large subunit (TerL, ORF 94) of the phage-packaging protein. Among the 39 functional genes, nine were related to DNA replication and nucleotide metabolism: the winged helix-turn-helix DNA-binding domain protein (ORF 76), replicative DNA helicase (ORF 78), DNA primase (ORF 80), putative DNA helicase (ORF 82), putative DNA-binding domain protein (ORF 83), ribonuclease H-like domain protein (ORF 88), DNA polymerase I (ORF 104), DNA methyltransferase (ORF 107), and putative DNA polymerase I (ORF 110). Three genes were identified as auxiliary metabolic genes (AMGs): rubredoxin-type fold protein (ORF 87), pyruvate phosphate dikinase (PPDK; ORF 99), and a transporter (ORF 103). The lysogeny-related gene was recombinase A (*recA*; ORF 85). Seventeen ORFs were involved in other functions, including coil-containing proteins (ORF 4, ORF 28, ORF 41, ORF 53, ORF 59, ORF 93, and ORF 98), transmembrane helix-containing proteins (ORF 15, ORF 74, and ORF 108), DUF550 domain-containing protein (ORF 7), XkdF (ORF 56), K^+^-dependent Na^+^/Ca^+^ exchanger (ORF 57), DUF5675 domain-containing protein (ORF 73), AAA family ATPase (ORF 75), putative protein-tyrosine phosphatase (ORF 101), and putative zinc- or iron-chelating domain-containing proteins (ORF 123). Eighty-seven ORFs were identified as hypothetical proteins. No tRNA, virulence, or resistance genes were detected in R21Y.

### 2.3. Taxonomy and Phylogeny of R21Y

**Figure 3:**
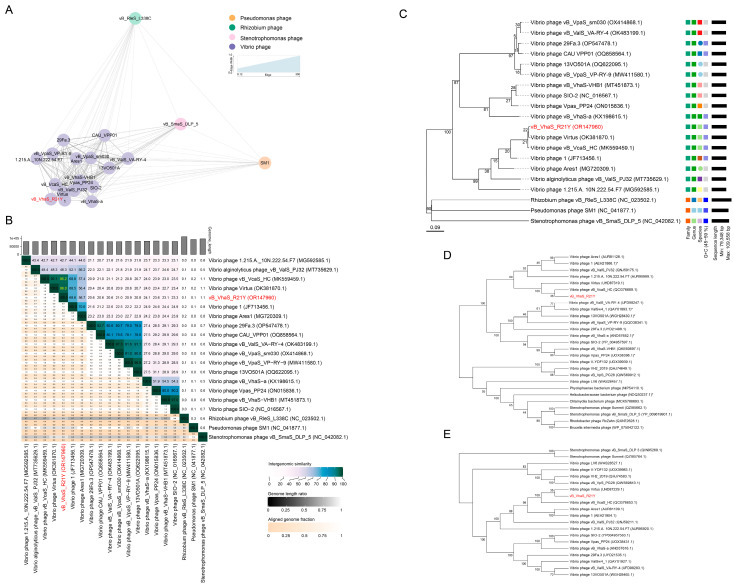
Evidence supporting the taxonomy and phylogeny of vB_VhaS_R21Y. (**A**) Protein-sharing network indicating evolutionary affinity among vB_VhaS_R21Y and its related phages sharing pairwise similarity scores of >1. Each node represents a phage genome and is colored according to its host taxonomy. Edges connecting pairwise phages from the same viral cluster determined by vConTACT2 are displayed. Thicker edges indicate a strong connection between the two phages. The valid names of existing phage genera are displayed. (**B**) Pairwise intergenomic distances/similarities among viral genomes for 20 phages as per the Virus Intergenomic Distance Calculator. (**C**) GBDP tree based on complete or partial genomes of compared phages using the web tool, VICTOR. (**D**) Neighbor-joining tree (1000 bootstraps) of vB_VhaS_R21Y and similar phages based on the amino acid sequences of major capsid protein as per MEGA 7. The asterisk indicates that the protein sequence has been manually annotated as the major capsid protein. (**E**) Neighbor-joining tree (1000 bootstraps) of vB_VhaS_R21Y and similar phages based on amino acid sequences of the terminase large subunit as per MEGA 7.

vConTACT2 detected 20 phages with similarity scores greater than one. Most of these phages (17) were isolated using *Vibrio* strains, while a few infected *Pseudomonas*, *Rhizobium*, and *Stenotrophomonas*. A gene content-based network using an edge-weighted spring-embedded layout confirmed that R21Y was most closely related to phages Virtus, vB_VcaS_HC, and 1 (Figure 3A, Table S2). Intergenomic similarities among the 20 phages showed that R21Y was closely related to vibriophages Virtus and vB_VcaS_HC, with similarities of 96.0% and 95.2%, respectively, suggesting that R21Y belongs to the same species as that of Virtus and vB_VcaS_HC (Figure 3B).

A phylogenetic tree was constructed based on pairwise comparisons of the amino acid sequences of R21Y and related phages detected by BLASTn and vConTACT2. Phages R21Y, Virtus, and vB_VcaS_HC formed a separate clade (Figure 3C), and phylogenetic trees based on the MCP and TerL proteins of these phages suggested that R21Y is most closely related to vB_VhaS_HC and Virtus (Figure 3D,E).

### 2.4. Lytic Ability of R21Y

PhageAI analysis provided strong evidence of R21Y’s virulence, with 97.85% probability. A PCR assay was conducted using the *MCP* of phage R21Y as a hallmark gene to ascertain the likelihood and stability of phage genome integration into the bacterial chromosome. Comparison with the positive and negative controls showed that six colonies were positive for the presence of lysogenic or pseudo-lysogenic bacteria (Figure S2B). However, the bacteria could not grow stably enough to be subcultured.

**Figure 4:**
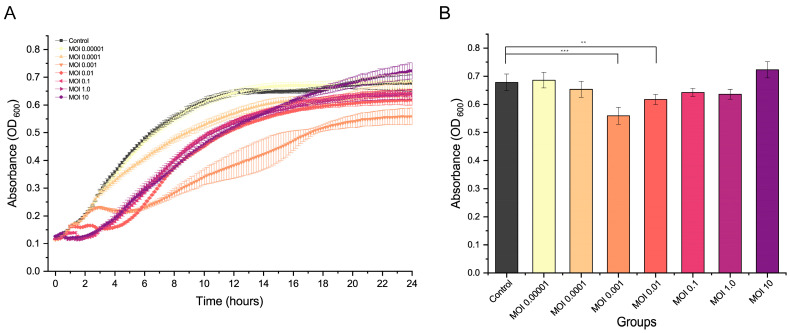
In vitro lysis of vB_VhaS_R21Y at different multiplicities of infection. (**A**) Growth curve for *V. harveyi* BVH1 infected by vB_VhaS_R21Y. (**B**) Absorbance (OD_600_) of the host at 24 h. Error bars indicate standard deviation among triplicate samples. Statistical significance is indicated by ** and *** at *p* < 0.01 and *p* < 0.001, respectively, compared with the control.

The killing curve showed that phages at multiplicities of infection (MOIs) of 0.00001 and 0.0001 did not significantly inhibit bacterial growth (Figure 4A). However, R21Y effectively infected and lysed the host within the first 2 h of incubation at MOIs of 0.1, 1.0, and 10, although phage R21Y did not significantly affect host growth after 24 h of incubation. Conversely, phage lysis negatively affected host growth at MOIs of 0.001 and 0.01 within 2–4 h of infection, whereas R21Y at MOIs of 0.001 (*p* < 0.001) and 0.01 (*p* < 0.01) significantly inhibited host growth compared with the control after 24 h of incubation (Figure 4B).

### 2.5. Thermal and pH Stability of R21Y

**Figure 5:**
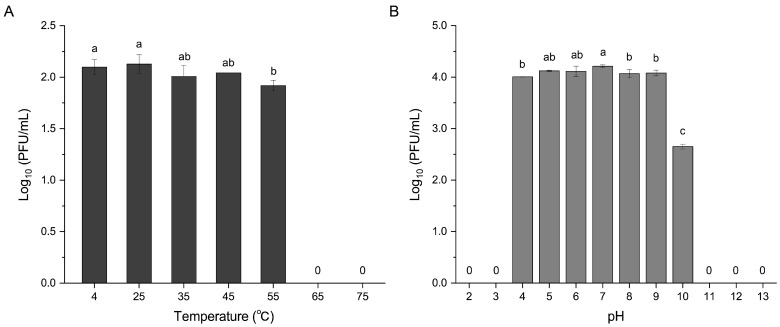
Stability profiles of phage vB_VhaS_R21Y over a range of temperatures and pHs. (**A**) Thermal stability profile. (**B**) pH stability profile. Error bars indicate standard deviation among triplicate samples. Letters on the columns indicate statistical significance at *p* < 0.05.

Stability tests showed that R21Y remained robust within 4–45 °C and pH 4–9 (Figure 5). However, when incubated at 55 °C and pH 10, the phage titer dropped significantly by several orders of magnitude. The phage titer rapidly decreased to zero when R21Y was incubated at temperatures >65 °C. R21Y was entirely inactive when incubated under extreme pHs of 2, 3, 11, 12, and 13.

## 3. Discussion

To date, 62 bacteriophages have been isolated against *V. harveyi*, including 33 siphoviruses, 15 myoviruses, and four podoviruses. Ten phages remain unknown (Table S3) [15,19,20,21,22,32,33,34,35,36,37,38,39,40,41,42,43,44,45,46,47,48,49,50,51,52,53,54]. Of these phages, only five vibriophages, VHML [15], 1 (VHS1) [19], VhCCS-19, VhCCS-21 [36], and VH-P [38], have been identified as temperate, while the rest are considered lytic. In this study, a novel *V. harveyi* phage, named R21Y, was isolated and characterized. The plaque diameter and burst size of R21Y were 0.69 ± 0.15 mm and 35 plaque-forming units (PFU) per cell, respectively. From a comparative analysis of phage R21Y with vibriophages VB_VhaS-R18L (2.0 mm, 54 PFU/cell) [53] and vB_VpS_PG28 (1.5–2.0 mm, 103 PFU/cell) [55], it is intuitively apparent that a larger burst size would result in a larger plaque size. i.e., the plaque size of *Vibrio* phages is positively correlated with burst size, which concurs with the classical model [56]. The latent periods of these phages ranged from 10 to 60 min, and the burst sizes were between 20 and 298 phage particles per cell. The latent period and burst size of R21Y were within the documented ranges for vibriophages. Lytic phages that rapidly lyse their hosts and release new phages are particularly valuable for phage therapy, and their swift action can potentially lead to the rapid recovery of aquatic animals infected with *Vibrio*. Therefore, phage R21Y has been proposed as a candidate for phage therapy because of its infection cycle. Additionally, R21Y has a distinct kinetic curve from vB_VcaS_HC [57], although they share similar genomic information (95.2% similarities) and the closest evolutionary distance. Phage R21Y has a shorter latent period (40 min vs. 1.5 h) and a smaller burst size (35 PFU/cell vs. 172 PFU/cell) than does vB_VcaS_HC. Several factors are suggested to influence burst size, such as the host metabolic activity and bacterial protein synthesis machinery [58,59]. The different physiological traits of these two phages with highly similar genomes may be due to different host organisms and phage–host interactions. More research is needed to understand the molecular mechanisms behind the substantial burst size. Compared with the most similar phages Virtus and vB_VcaS_HC, R21Y has a smaller burst size and weaker infectivity, indicating a positive correlation between burst size and infectivity. Moreover, *Stenotrophomonas* phage vB_SmaS_DLP_5 [60] was clustered together with R21Y in the protein-sharing network. This indicates that R21Y may share similar protein functions with virus vB_SmaS_DLP_5 which belongs to the genus *Delepquintavirus*. vB_VcaS_HC is considered a new species of the genus *Delepquintavirus*. These finding suggest that R21Y may be a member of the genus *Delepquintavirus*.

The phage’s tail fiber “tip” domain or receptor-binding domain mediates the interaction between bacteriophages and their host. The host range of a phage is primarily determined by phage tail fibers, which initially mediate specific recognition and adsorption by susceptible bacteria [61]. However, no genes exist in the R21Y genome that correspond to the “tail fiber”, despite R21Y’s narrow spectrum and highly specific host range. Regarding their application, phages with broad host ranges can more effectively control diverse bacterial strains. However, phages with narrow host ranges can be added to phage cocktails to combat a wide range of bacteria and reduce the development of resistance [62]. Additionally, phages with narrower host ranges can help protect the host’s local microbiota during treatment, where specific *Vibrio* are prevalent in the environment.

R21Y exhibited two AMGs: rubredoxin-type fold protein and PPDK. Rubredoxin-type fold protein is a common iron–sulfur protein in many living systems. It serves as a small electron transfer protein in various biological reactions that produce energy and can act as an oxygen oxidoreductase, safeguarding bacterial cells by blocking creation of reactive oxygen species (ROS) in microaerophilic environments [63]. In aquaculture, environmental hypoxia and hyperoxia and the use of ozonization lead to increased oxidative stress [64]. Expression of rubredoxin-type fold protein may protect the host from external ROS, which is more conducive to phage reproduction. PPDK is responsible for the reversible conversion of phosphoenolpyruvate to pyruvate, a crucial step in the Embden–Meyerhof–Parnas pathway. This pathway is the primary pathway for glucose degradation and energy production. PPDK exists in bacteria, protists, and plants but has been found in only a few *Vibrio* siphoviruses [35,48,53,57]. When R21Y infects host cells, expression of the PPDK gene may aid the host’s carbohydrate metabolism, resulting in more energy for the phage progeny. Additionally, 16 uncategorized ORFs showed a wide range of functions, including serine protease XkdF, which is frequently found in phage genomes. The function of virally encoded serine proteases remains unknown, but they are strongly expressed during the late stage of viral infection, suggesting their potential role in virion assembly or maturation [65]. The K^+^-dependent Na^+^/Ca^+^ exchanger gene is a key determinant of Ca^2+^ signaling and homeostasis, especially in environments in which ion concentrations undergo large changes [66]. A putative protein-tyrosine phosphatase gene is highly conserved in the Orthopoxvirus family, suggesting its importance in viral replication or pathogenesis [67]. Expression of these genes may be useful for phage replication assembly in phage therapy.

R21Y has a potential lysogeny-related gene, *recA* (ORF 85). Through cleaved CI molecules, *recA* induced phage λ to switch from the lysogenic to the lytic state in an *Escherichia coli* host in response to DNA damage (via ultraviolet irradiation) [68]. *recA* has also been found in phages VHS1 [41], vB_VcaS_HC, and Virtus. VHS1 can integrate into its host, *V. harveyi* (VH)1114, to form lysogenic bacteria with superinfection immunity on the lysogenic test. However, VHS1 lysogens of VH1114 were unstable and spontaneously released VHS1 in liquid cultures. Li et al. found that vB_VcaS_HC did not form lysogen in its host, *V. campbellii* 18, according to the lysogeny test and PCR assay. Virtus has not been studied in this way; hence, its lysogenic capacity is unknown. In our study, R21Y could lysogenize or pseudo-lysogenize only 6.38% of host bacterial colonies; however, these bacteria could not be stably passaged, likely owing to spontaneous induction of R21Y, which can reproduce, lyse, and kill the host bacteria, as can VHS1. Multiple sequence alignments of the recA proteins of four bacteriophages showed a high sequence identity of 98.88% (Figure S3). Thus, the lysogenic capacities of the phages differ, likely because of differences in other genes with unknown functions. Therefore, R21Y is a lytic phage and cannot undergo stable lysogenization, which is consistent with the requirements of phage therapy.

In vitro lysis test results showed that phage R21Y was ineffective in eliminating host bacteria *V. harveyi* BVH1 at an MOI < 0.001. This may be due to a lack of phages capable of lysing a substantial amount of *Vibrio* in a short period; however, host bacteria can become resistant to phage R21Y over time. MOIs of 0.001 and 0.01 were the best phage/bacteria ratios and had the best lysis effect on the host; thus, these doses are recommended for practical applications of phage therapy. The dose may become less effective if large phages (MOIs 1.0 and 10) are injected into *Vibrio* outbreak ponds; therefore, the phage should be injected at an appropriate level during phage therapy. In the future, rigorous testing is still indispensable for practical applications. Additionally, R21Y maintained high lytic activity over varying environmental conditions (4–55 °C and pH 4–10). R21Y showed better thermal tolerance than vB_VhaM_pir03 (45 °C) [21], VPMCC5 (45 °C) [50], vB_VhaS_MAG7 (45 °C) [54], V-YDF132 (50 °C) [49], and vB_VhaS-R18L (50 °C) [53] and greater acid tolerance than V-YDF132 (pH 5–11) and vB_VhaS-R18L (pH 6–11). Thus, R21Y can remain stable and exert bactericidal activity in practical applications, suggesting that it is a promising candidate for biological control of *V. harveyi* disease.

In this study, we clarified the physiological, genomic, and phylogenetic characteristics of lytic *Vibrio* phage R21Y. R21Y specifically infected host bacteria, and no antibiotic resistance or virulence genes were detected in the R21Y genome. These results suggest that R21Y may be a safe biocontrol agent for controlling vibriosis. Furthermore, R21Y may be a candidate for phage therapy owing to its stability over a wide range of temperatures (up to 55 °C) and pHs of 4–10. Some AMGs have been identified in R21Y, including a rubredoxin-type fold protein and PPDK, which may be beneficial for assembling and releasing progeny phages. More research is needed to advance the applications of R21Y in actual phage therapy.

## 4. Materials and Methods

### 4.1. Phage Isolation and Purification

The host strain *V. harveyi* BVH1 was purchased from the National Pathogen Collection Center for Aquatic Animals, Shanghai Ocean University (Shanghai, China). *V. harveyi* BVH1 was grown in a seawater-based liquid medium amended with 30 g/L sea salts, 10 g/L peptone, and 3 g/L yeast extract and then incubated at 28 °C with agitation at 160 rpm/min. Phage-containing water samples were collected from a scallop farm at the Bay of Ellen, Rongcheng, Shandong Province, China (122°34′14.54″ W, 37°10′15.58″ E) and filtered through 0.22-µm sterile filters (Millipore, Bedford, MA, USA). To increase the probability of isolating phages, the filtered water was inoculated with exponentially growing host culture and incubated overnight. After incubation, the mixed culture solution was centrifuged (10,000× *g*, 10 min, 4 °C) and filtered through 0.22-µm sterile filters to remove residual cells and impurities. The filtered culture was used for phage isolation, using the double-agar layer method as per Clokie et al. [69]. After plaques emerged, an individual plaque was collected, dissolved in SM buffer (5.8 g/L NaCl, 2 g/L MgSO_4_, 50 mL 1M Tris-HCl pH 7.5 and 2% gelatin, 1 L deionized H_2_O) and purified through at least five cycles.

### 4.2. Preparation of High-Titer Phage Suspensions

To obtain high-titer phages, purified phages were inoculated into exponentially growing host culture and amplified to 1 L. After cell lysis, the culture was treated with 2 mg/L DNase, 2 mg/L RNase, and 20 g/L NaCl to separate the phage particles from host cell debris and then centrifuged at 10,000× *g* for 10 min at 4 °C. The supernatant was filtered through 0.22-µm membranes and precipitated with polyethylene glycol 8000 (10% [wt/vol]) overnight at 4 °C. After centrifugation at 10,000× *g* for 60 min at 4 °C, the phage precipitate was resuspended in SM buffer and further purified via CsCl equilibrium gradient centrifugation (200,000× *g*, 4 °C, 24 h) using an Optima L-100 XP ultracentrifuge (Beckman Coulter, Brea, CA, USA). The visible phage band was extracted and desalted through 30-kDa super filters (Millipore, Bedford, MA, USA).

### 4.3. Transmission Electron Microscopy

Phage morphology was examined using the single-negative staining method via transmission electron microscopy. Briefly, 10 µL of high-titer phage was adsorbed onto a carbon-coated formvar grid for 30 min in the dark. Then the phage sample was stained with 1% phosphotungstic acid for 1 min and air dried. The grids were examined with a JEM-2100 transmission electron microscope (JEOL Ltd., Akishima, Tokyo, Japan) at 80 kV. Images were collected using the CCD image transmission system (Gatan Inc., Pleasanton, CA, USA). Phage head and tail dimensions were measured using ImageJ v2.35 (http://imagej.net/ [accessed on 17 March 2023]) for at least five individual phage particles [70].

### 4.4. One-Step Growth Curve

A one-step growth experiment was performed to determine the phage’s life cycle [71]. Briefly, 1 mL of early-phase host culture was infected with the phage at an MOI of 0.01 and allowed to adsorb for 15 min at room temperature in the dark. Mixtures were then centrifuged at 8000× *g* for 10 min to remove unabsorbed phage particles. After discarding the supernatant, the pellet was washed and resuspended twice in 1 mL of SM buffer, transferred to 50 mL of 2216E medium, and then incubated at 28 °C in the dark with shaking at 160 rpm/min. Subsamples for the phage titer were taken at 20-min intervals for 140 min with three biological replicates and determined by the double-layer agar method.

### 4.5. Host Range

The host range of the phage was determined using a spot assay [72]. Briefly, 41 *Vibrio* strains from 21 *Vibrio* species (*V. harveyi*, *V. alginolyticus*, *V. azureus*, *V. campbellii*, *V. chagasic*, *V. diazotrophicus*, *V. fortis*, *V. hyugaensis*, *V. inhibens*, *V. mytili*, *V. natriegens*, *V. neocaledonicus*, *V. owens*, *V. parahaemolyticus*, *V. plantisponsor*, *V. ponticus*, *V. rotiferianus*, *V. tubiashii*, *V. variabilis*, *V. xiii*, and *V. ziniensis*) were purchased from the National Pathogen Collection Center for Aquatic Animals, Shanghai Ocean University (Shanghai, China) and the Institute of Marine Microbes and Ecospheres, Xiamen University (Xiamen, China). Each exponentially growing bacterial culture was mixed with molten soft agar (0.5% [wt/vol]), then immediately poured onto a solid agar plate (1.5% [wt/vol]). After solidification of the agarose plates, 5 µL of phage lysate was spotted onto the bacterial lawn, and the agar plates were incubated at 28 °C for >24 h. The presence of plaques within the bacterial lawn was assessed to determine whether the phage had infected the host bacterium.

### 4.6. Lysogeny Test

The phage’s ability to lysogenize its trapping host was tested by isolating bacterial cells from the plaque [73]. Approximately 5 µL of the phage solution was spotted onto the host lawn to allow plaque formation. The phage plaque was then removed and stored in 1 mL phosphate-buffered saline (PBS). The suspension was centrifuged at 8000× *g* for 10 min, and the resulting cell pellets were washed twice with PBS to eliminate extracellular phages and then resuspended in 1 mL of freshly prepared 2216E medium. Growing colonies were obtained by plating aliquots (100 µL) of 1:1000 cell suspensions on 1.5% 2216E agar plates and assessed for lysogeny via colony PCR with primers targeting the phage gene encoding *MCP* (Table S4). PCR reactions were performed with a 25-µL reaction mixture containing 12.5 µL *Premix Taq* (Takara Bio Inc., Kusatsu, Shiga, Japan), 1 µL of each primer (10 mM), 1.0 µL template DNA, and 9.5 µL DNase/RNase-free distilled water. Cycling conditions were preheating at 95 °C for 10 min, followed by 30 cycles of denaturation at 95 °C for 30 s, annealing at 57 °C for 30 s, and extension at 72 °C for 1 min, with a final elongation step at 72 °C for 5 min. The phage DNA served as the template for the positive control, and wild-type *V. harveyi* BVH1 was used as the negative control. The PCR amplification products were detected via 1.0% agarose gel electrophoresis, and positive colonies were further passaged three times to verify the stability of the lysogeny.

### 4.7. Lytic Efficacy

In vitro lysis assays of the phage against *V. harveyi* BVH1 were conducted in a sterile 96-well plate using a Synergy H1 microplate reader (BioTek Instruments Inc., Winooski, VT, USA). Briefly, early-log-phase host bacterial culture was loaded into a well and infected with the phage at MOIs of 0.00001, 0.0001, 0.001, 0.01, 0.1, 1.0, and 10. Host bacteria without phages served as the control. The plate was placed in the microplate reader and incubated at 28 °C with orbital shaking. The OD_600_ of the cultures was measured every 10 min for 24 h. The assay was performed six times per treatment, and the data from each group at 24 h were compared with those of the control group using an independent sample t-test performed with SPSS software (IBM SPSS Statistics 25, IBM, Armonk, NY, USA).

### 4.8. Thermal and pH Stability

To test the effect of environmental factors on the phage, thermal and pH stability tests were conducted as previously reported by Kim et al. with minor modifications [74]. For the thermal stability test, 1 mL of phage solution (10^5^ PFU/mL) was incubated statically at 4, 25, 35, 45, 55, 65, and 75 °C for 1 h. After incubation, the phage titer was determined using the double-layer plate method. To evaluate pH stability, phage samples at 10^7^ PFU/mL were inoculated into SM buffer with gradient pH values (2–13) achieved by adjustment with 1 M NaOH and 1 M HCl. The solutions were statically incubated for 1 h at room temperature, and the phage titers were determined using the double-layer agar technique. The experiment was conducted in triplicate, and statistical differences were assessed using one-way analysis of variance followed by Duncan’s multiple range test at *p* < 0.05.

### 4.9. DNA Extraction

Phage genomic DNA was extracted using a standard phenol-chloroform extraction protocol [75]. To eliminate the potential for free host DNA and RNA contaminants, the purified phages were treated with protease K (100 mg/mL), sodium dodecyl sulfate (10% [wt/vol]), and EDTA (0.5 mol/mL; pH 8.0) for 3 h at 55 °C. The phage sample was then extracted and purified using phenol-chloroform-isoamyl alcohol (25:24:1 [vol/vol]) and chloroform-isoamyl alcohol (24:1 [vol/vol]) to remove any impurities. The phage DNA from the resulting supernatant was sequentially precipitated with isopropanol and stored overnight at −20 °C. The precipitate was washed twice with 70% ethanol, air dried, dissolved in sterile Tris-EDTA buffer (10 mM Tris-HCl and 1 mM EDTA [pH 8.0]), and stored at −80 °C before sequencing.

### 4.10. Genome Sequencing and Annotation

The phage genome was paired-end sequenced using high-throughput Illumina NovaSeq. Velvet software (v1.2.03) was used for genome assembly after removing low-quality reads [76]. Phage termini and packaging mechanisms were predicted with the PhageTerm tool (v3.0.1) [77]. GeneMarkS (v4.32) was used to identify ORFs within the genome [78]. The tRNA genes were searched using tRNAScan-SE v2.0 [79]. The Eeayfig tool was used for genome visualization [80]. Predicted genes were annotated manually using BLASTp [81], CD-search [82], and RAST [83]. The absence of potentially toxic genes and antibiotic-resistance determinants was confirmed via comparison with the Virulence Factors of Pathogenic Bacteria [84] and Comprehensive Antibiotic Resistance Databases [85]. To determine the life strategy of the phage, genome-based life cycle classification was performed using an AI-driven software platform (https://phage.ai/ [accessed on 17 June 2023]) and PCR assay (*MCP* gene of phage R21Y). The annotated genome sequence of the phage was deposited in the NCBI GenBank database (accession number OR147960).

### 4.11. Taxonomic Network and Phylogenetic Analysis

The dataset comprising 4516 genomes (containing 468,105 proteins) of prokaryotic viruses from NCBI Refseq (v207) and phage genomes similar to R21Y from the NCBI nucleotide database were downloaded and compared with the phage using vConTACT2, which identifies viral clusters [86]. For clarity, viruses showing similarity scores of >1 to the phage were exported for subsequent analysis. A visual representation of the protein-sharing network was generated using Cytoscape (v3.9.1) [87]. The edge-weighted spring-embedded model was selected, with viral similarity scores serving as the weight. This arrangement ensured that viral genomes sharing more viral protein clusters would be positioned closer together. Intergenomic similarities between the phage and related phages were calculated based on nucleotide data using the Virus Intergenomic Distance Calculator [88]. Finally, the phage was taxonomically classified using the traditional algorithm used by the International Committee on Taxonomy of Viruses through this tool.

To gain insight into the phylogenetic relationships among phages, complete genomes were submitted to the VICTOR server (https://ggdc.dsmz.de/victor.php [accessed on 15 September 2023]), and the genome BLAST distance phylogeny method was applied, using settings recommended for prokaryotic viruses [89]. The resulting tree was rooted at the midpoint with branch support inferred from 100 pseudo-bootstrap replicates. The phage MCP and TerL sequences were used to construct the neighbor-joining phylogenetic trees using MEGA 7 with 1000 bootstraps [90].

## Figures and Tables

**Table 1:** Host range of phage vB_VhaS_R21Y.

Species	Strain	Strain Type	Infectivity
*V. harveyi*	BVH1	Pathogenic	+
	20150916-2	Pathogenic	−
	Chan01	Nonpathogenic	−
	CGMCC 1.1593 ^T^	Nonpathogenic	−
	LMG 4044 ^T^	Nonpathogenic	−
*V. alginolyticus*	ATCC 17749 ^T^	Pathogenic	−
	HJ-1	Nonpathogenic	−
	JL2674	Nonpathogenic	−
	ZWCM4003	Nonpathogenic	−
*V. azureus*	JL1214	Nonpathogenic	−
	JL3560	Nonpathogenic	−
	JL3577	Nonpathogenic	−
*V. campbellii*	HJ-2	Nonpathogenic	−
	JL2671	Nonpathogenic	−
	JL3506	Nonpathogenic	−
	JL3507	Nonpathogenic	−
*V. chagasic*	JL3518	Nonpathogenic	−
*V. diazotrophicus*	JCM 21185	Pathogenic	−
*V. fortis*	JL3515	Nonpathogenic	−
	JL3565	Nonpathogenic	−
	JL3678	Nonpathogenic	−
*V. hyugaensis*	JL3710	Nonpathogenic	−
*V. inhibens*	JL3707	Nonpathogenic	−
*V. mytili*	JL3548	Nonpathogenic	−
*V. natriegens*	WPAGA4	Nonpathogenic	−
*V. neocaledonicus*	JL3539	Nonpathogenic	−
	JL3558	Nonpathogenic	−
	JL3563	Nonpathogenic	−
	JL3703	Nonpathogenic	−
*V. owens*	JL2663	Nonpathogenic	−
	JL3650	Nonpathogenic	−
	JL2918	Nonpathogenic	−
*V. parahemolyticus*	Chan02	Nonpathogenic	−
	ATCC 17802 ^T^	Pathogenic	−
*V. plantisponsor*	DSM 21026	Pathogenic	−
*V. ponticus*	JL4287	Nonpathogenic	−
*V. rotiferianus*	JL3557	Nonpathogenic	−
*V. tubiashii*	JL3833	Nonpathogenic	−
*V. variabilis*	JL3468	Nonpathogenic	−
*V. xiii*	JL2919	Nonpathogenic	−
*V. ziniensis*	ZWAL4003	Nonpathogenic	−

“+” indicates infected; “−” indicates uninfected; the superscript letter “T” represents type strain.

## Data Availability

Annotated genomes of *Vibrio harveyi* phage vB_VhaS_R21Y were deposited in GenBank under accession number OR147960.

## Supplementary Materials

The following supporting information can be downloaded at: https://www.mdpi.com/article/10.3390/ijms242216202/s1.
