# Supplementary material for: Characterization of a Vibriophage Infecting Pathogenic Vibrio harveyi
Source: Int J Mol Sci. 2023 Nov 11;24(22):16202. doi: 10.3390/ijms242216202 (PMC10671443; doi:10.3390/ijms242216202)
Supplement: Supplementary file 1 [file ijms-24-16202-s001.zip › Lyy-phage vB_VhaS_R21Y-Supplementary Materials-20231002.pdf]

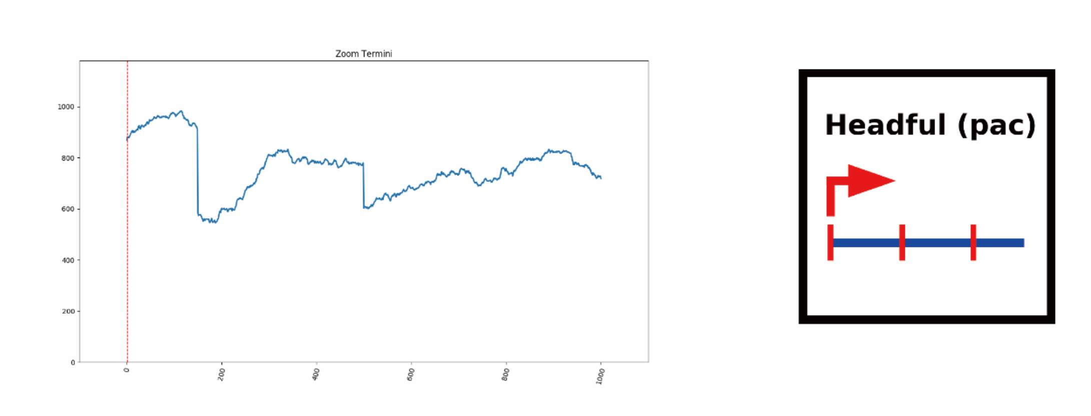

Figure S1. Termini Analysis of vB_VhaS_R21Y by Phageterm.

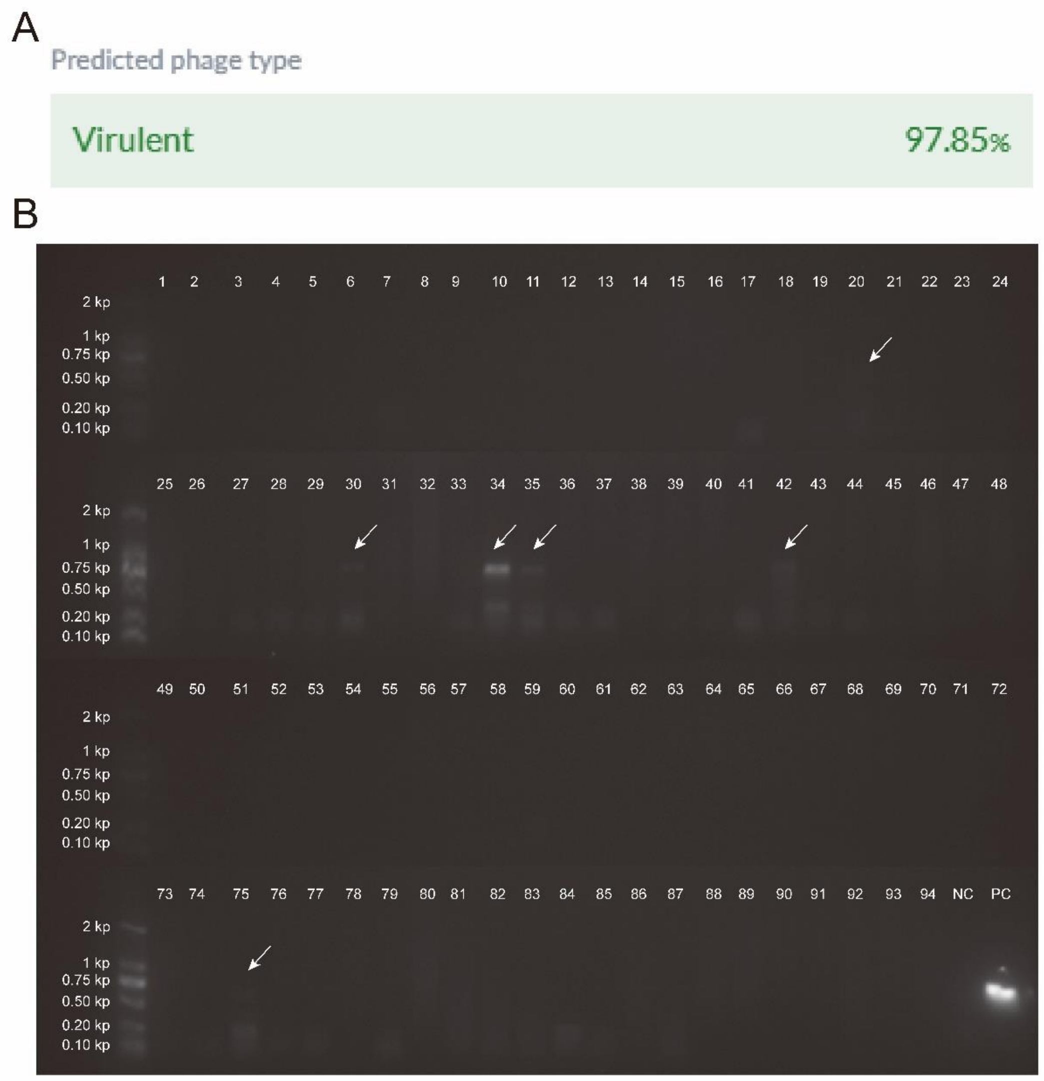

Figure S2. Lifestyle Analysis of vB_VhaS_R21Y. (A) Bacteriophage Life Cycle Recognition of vB_VhaS_R21Y by PhageAI. (B) Lysogeny Test of vB_VhaS_R21Y base on PCR with Specific Primers. Arrows indicated positive clones of lysogenic or pseudolysogenic bacteria. NC: negative control. PC: positive control.

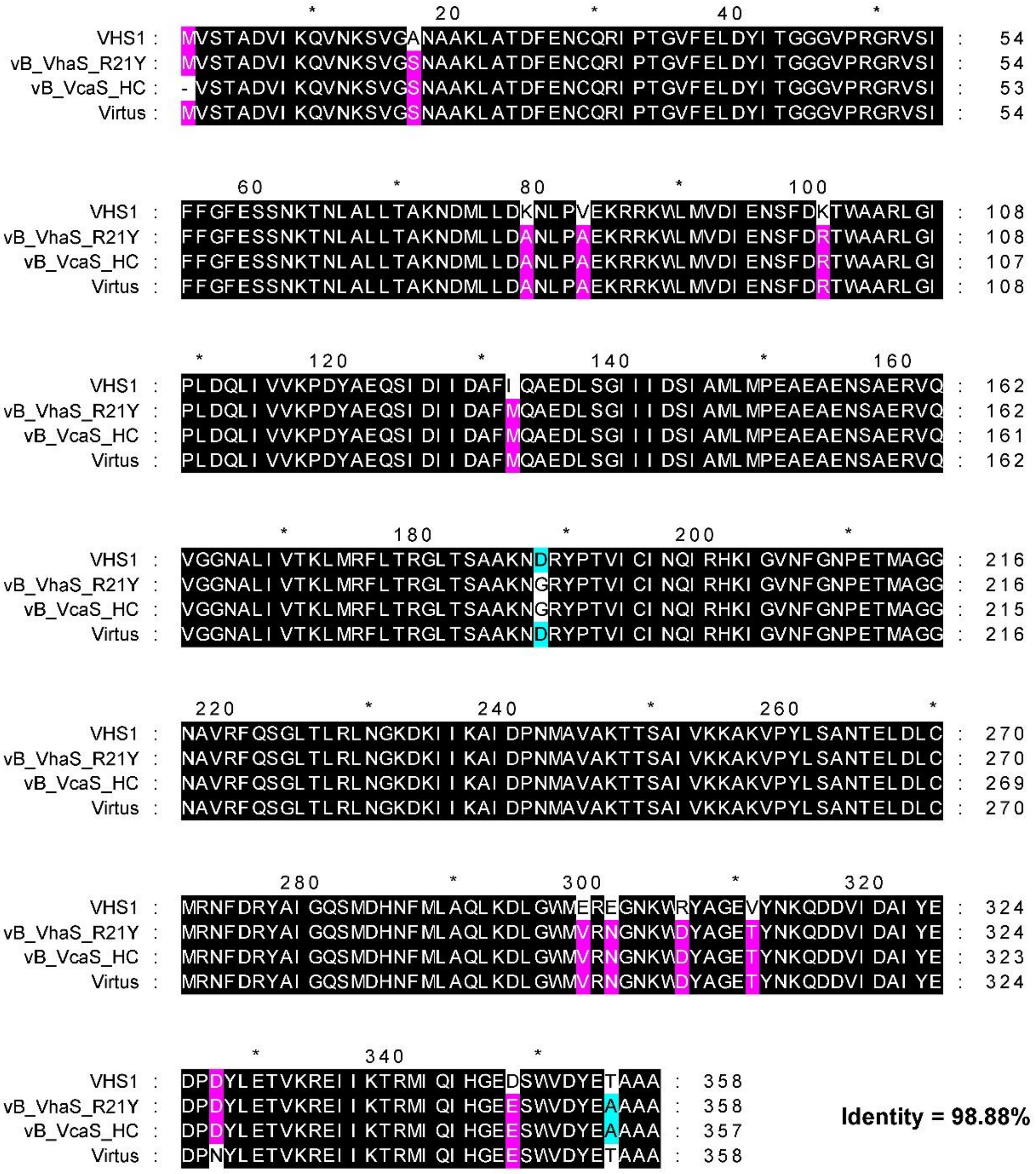

Figure S3. Multiple Sequence Alignments of RecA Proteins of Four Bacteriophages.

Table S1. The Detected ORFs of vB_VhaS_R21Y, including the Length and the Putative Product.
Data in EXCEL.

Table S2. The Edge-score of vB_VhaS_R21Y by vConTACT2 Compared with Related Phages in the ProkaryoticViralRefSeq207 Database and NCBI Nucleotide Database.

Data in EXCEL.

Table S3. Isolated Bacteriophages against *V. harveyi*.

Phage	Morphology			Genome		One-step curve		Stability		Public year	Country
	Category	Capsid diameter (nm)	Tail length (nm)	Size (bp)	ORF	Latent period (min)	Burst size (PFU/cell)	Temperature (℃)	pH		
VHML[#]	Myovirus									2000	Australia
VHS1[#]	Siphovirus	66 ± 3	153 ± 10	81,509	125			37-60	3-11	2005	Thailand
A	Siphovirus	40 - 45	60							2006	India
Viha1	Siphovirus	56 ± 5	176 ± 9	94,000						2007	India
Viha2	Siphovirus	53 ± 3	200 ± 18	94,000							
Viha3	Siphovirus	56 ± 5	211 ± 22	70,000							
Viha4	Myovirus	114 ± 9	192 ± 22	85,000							
Viha5	Siphovirus	92 ± 6	175 ± 19	83,000							
Viha6	Siphovirus	Elongated	126 ± 12	60,000							
Viha7	Siphovirus	58 ± 3	194 ± 16	44,000							
Viha8	Siphovirus									2007	India
Viha9	unknown										
Viha10	Siphovirus	40 - 45	60								
Viha11	unknown										
VhCCS-01	Siphovirus	60	100							2010	Australia
VhCCS-02	Siphovirus	60	100								
VhCCS-04	Siphovirus	60	100								
VhCCS-06	Siphovirus	60	100								
VhCCS-17	Siphovirus	60	100								
VhCCS-19[#]	Myovirus	60	100								
VhCCS-20	Siphovirus	60	100								
VhCCS-21[#]	Myovirus	60	100								

PW2	Siphovirus	50 ± 3.8	136 ± 6.2			30	78			2010	Thailand
φVh1	Siphovirus	80 ± **7**	170	85,000						2011	India
φVh2	Siphovirus	60 ± 12	130	57,000							
φVh3	Podovirus	72 ± 5	27	64,000							
φVh4	Siphovirus	115 ± 4	329	107,000							
VH-P[#]	Podovirus	70–80								2011	Thailand
SIO-2	Siphovirus	81±4	209 ± 16	80,598	116	45-60	60	4-60	3-10	2012	USA
VHP1	unknown									2014	India
VHP2	unknown										
VHP3	unknown										
VHP4	unknown										
VHP5	unknown										
VHP6a	unknown										
VHP6b	Siphovirus	96	145	78,081	104						
VHP7	unknown										
Vi ha 6	Myovirus	70.8	152							2014	India
Vi ha 15	Myovirus	84	180.6								
Vi ha 19	Myovirus	94.4	186.1								
Vi ha 21	Myovirus	78.9	197.3								
Vi ha 32	Myovirus	107.1	221.4								
Vi ha 68	Siphovirus	62.8	277.1								
VH7D*	Myovirus	75	155	246,964	378					2015	China
VhKM4	Myovirus	107 ± 4.5	105.2 ± 3.7			60	52			2017	Japan
vB_VhaS-a	Siphovirus			82,000						2017	Australia
vB_VhaS-tm	Siphovirus			59,000							
VHM1	Myovirus	66 ± 5	180 ± 5			20	84	4-50	4-10	2017	India

VHM2	Myovirus	68 ± 5	147 ± 7			10	60	4-50	4-10		
VHS1	Siphovirus	47 ± 7	227 ± 10			20	76	4-50	4-10		
vB_VhaM_pir03*	Myovirus	131.79 ± 6.14	222.03 ± 6.94	286,284	334	40	75	4-45		2020	Greece
VB_VhaP_Vh-5	Podovirus	50 ± 1.2	13 ± 1.1	42,485	49					2021	China
VB_VhaP_Vh-8	Myovirus	68 ± 1.2	101 ± 1.1	87,255	150						
vB_VhaS_PcB-1G	Siphovirus	68	125	48,719	80	10	210			2021	China
Virtus	Siphovirus	70 ± 05	220 ± 10	82,960	127	40	3200	4-55	4-10	2022	Greece
No-name	unknown					40	48	40-70	4-9	2022	India
VPMCC5	Podovirus	40.5 ± 4.1	13.0 ± 1.7	48,938	71	10	20	4-45	3-9	2022	India
V-YDF132	Siphovirus	69	160	84,375	115	25	298	37-50	5-11	2022	China
φLV6	Siphovirus	73	191	79,862	107					2023	India
vB_VhaS-R18L	Siphovirus	88.6 ± 2.2	225	80,965	118	40	54	4-50	6-11	2023	China
vB_VhaS_MAG7	Siphovirus	75 ± 4	93 ± 8	49,315	76	20	1393	4-45	4-10	2023	Greece
vB_VhaS_R21Y	Siphovirus	73.31 ± 2.09	205.55 ± 0.75	82,795	126	40	35	4-55	4-10	2023	China

#Temperate phage; *Jumbo phage.

Table S4. Primer Information for the Lysogeny Test of vB_VhaS_R21Y.

Primer name	Sequence (5'->3')	Template strand	Length	Start	Stop
R21Y_mcp_F	CGTGTAACGGGTGCGTTTTC	Plus	20	379	398
R21Y_mcp_R	TAACGTTCGCCTGTTCGTCT	Minus	20	928	909
